# Poly[ethane-1,2-diammonium tetra-μ-chlorido-cadmate(II)]

**DOI:** 10.1107/S1600536809002025

**Published:** 2009-01-23

**Authors:** Abdellatif Lamhamdi, Elmiloud Mejdoubi, Karla Fejfarová, Michal Dušek, Brahim El Bali

**Affiliations:** a Department of Chemistry, Faculty of Sciences, University Mohammed 1st, Po Box 717, 60000 Oujda, Morocco; b Institute of Physics, Na Slovance 2, 182 21 Praha 8, Czech Republic

## Abstract

The framework of the title compound, {(NH_3_CH_2_CH_2_NH_3_)[CdCl_4_]}_*n*_, is built upon layers parallel to (100) made up from corner-sharing [CdCl_6_] octa­hedra. NH_3_CH_2_CH_2_NH_3_^2+^ cations are situated between the layers and are linked to the layers *via* an N—H⋯Cl hydrogen-bonding network. The Cd atom is located on an inversion centre and the coordination environment is described as highly distorted octa­hedral.

## Related literature

Isotypic structures have been reported by Berg & Sotofte (1976 ▶), (NH_3_CH_2_CH_2_NH_3_)[PdCl_4_]; Birrell & Zaslow (1972 ▶), (NH_3_CH_2_CH_2_NH_3_)[CuCl_4_]; Tichý *et al.* (1978 ▶), (NH_3_CH_2_CH_2_NH_3_)[MnCl_4_]; Skaarup & Berg (1978 ▶), (NH_3_CH_2_CH_2_NH_3_)[NiCl_4_]. For the structures of related compounds, see: Woode *et al.* (1987 ▶), CdCl_2_^.^CH_5_N_2_S·H_2_O; Furmanova *et al.* (1996 ▶), CdCl_2_·CO(NH_2_)_2_; Wang *et al.* (1993 ▶), CdCl_2_·NH_2_NHCONH_2_; Cavalca *et al.* (1960 ▶), CdCl_2_^.^2(C_2_H_5_N_3_O_2_). For crystallographic background, see: Becker & Coppens (1974 ▶).
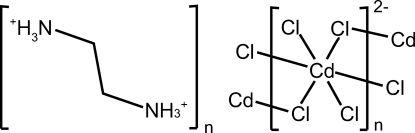


## Experimental

### 

#### Crystal data

(C_2_H_10_N_2_)[CdCl_4_]
*M* *_r_* = 316.3
Monoclinic, 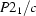

*a* = 8.6205 (5) Å
*b* = 7.3425 (8) Å
*c* = 7.2937 (7) Å
β = 92.791 (6)°
*V* = 461.11 (7) Å^3^
*Z* = 2
Mo *K*α radiation
μ = 3.45 mm^−1^
*T* = 298 K
0.27 × 0.13 × 0.08 mm

#### Data collection

Oxford Diffraction Gemini diffractometer with Atlas CCD detector
Absorption correction: analytical [implemented in *CrysAlis RED* (Oxford Diffraction, 2008 ▶), according to Clark & Reid (1995 ▶)] *T*_min_ = 0.605, *T*_max_ = 0.841
6603 measured reflections
960 independent reflections
899 reflections with *I* > 3σ(*I*)
*R*_int_ = 0.023

#### Refinement

*R*[*F* ^2^ > 2σ(*F* ^2^)] = 0.010
*wR*(*F* ^2^) = 0.027
*S* = 1.08
960 reflections
44 parameters
H-atom parameters constrained
Δρ_max_ = 0.13 e Å^−3^
Δρ_min_ = −0.13 e Å^−3^

### 

Data collection: *CrysAlis CCD* (Oxford Diffraction, 2005 ▶); cell refinement: *CrysAlis RED* (Oxford Diffraction, 2008 ▶); data reduction: *CrysAlis RED*; program(s) used to solve structure: *SIR2002* (Burla *et al.*, 2003 ▶); program(s) used to refine structure: *JANA2006* (Petříček *et al.*, 2007 ▶); molecular graphics: *DIAMOND* (Brandenburg & Putz, 2005 ▶); software used to prepare material for publication: *JANA2006*.

## Supplementary Material

Crystal structure: contains datablocks global, I. DOI: 10.1107/S1600536809002025/wm2213sup1.cif

Structure factors: contains datablocks I. DOI: 10.1107/S1600536809002025/wm2213Isup2.hkl

Additional supplementary materials:  crystallographic information; 3D view; checkCIF report

## Figures and Tables

**Table 1:** Selected bond lengths (Å)

Cd1—Cl1	2.6427 (5)
Cd1—Cl1^i^	2.6471 (5)
Cd1—Cl2	2.5585 (4)

Symmetry code: (i) 
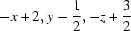
.

**Table 2:** Hydrogen-bond geometry (Å, °)

*D*—H⋯*A*	*D*—H	H⋯*A*	*D*⋯*A*	*D*—H⋯*A*
N1—H3⋯Cl1^ii^	0.87	2.35	3.2123 (14)	173
N1—H4⋯Cl2^iii^	0.87	2.46	3.2824 (15)	157
N1—H5⋯Cl2	0.87	2.34	3.2075 (12)	172

Symmetry codes: (ii) 
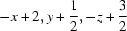
; (iii) 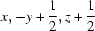.

**Fig. 1.**
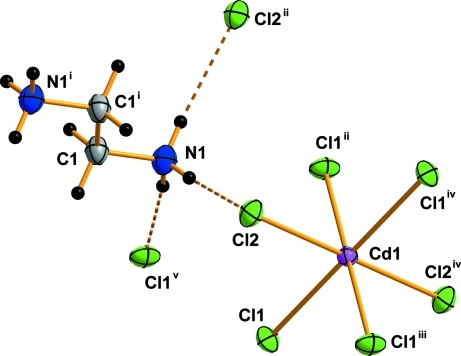
Part of the structure of (NH3CH2CH2NH3)[CdCl4]. Displacement ellipsoids are drawn at the 50% probability level. Hydrogen bonds are represented by dashed lines. [Symmetry codes: (i) 1 - x, 1- y, 2 - z; (ii) x, 0.5 - y, 0.5 + z; (iii) 2 - x, -0.5 + y, 1.5 - z; (iv) 2 - x, -y, 2 - z; (v) 2 - x, 0.5 + y, 1.5 - z]

**Fig. 2.**
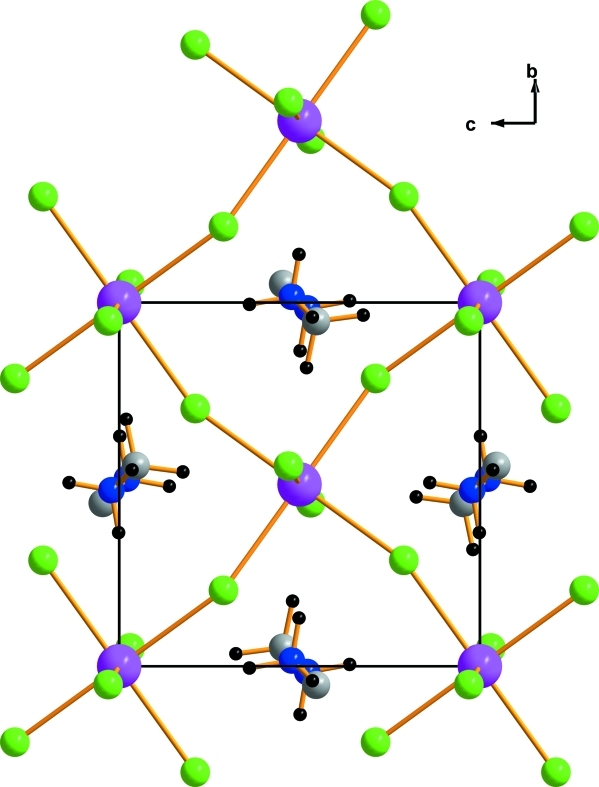
Packing of (NH3CH2CH2NH3)[CdCl4] viewed along a. Color code: Pink balls (Cd), green balls (Cl), grey balls (C), blue balls (N), black balls (H).

## supplementary crystallographic information

### Comment

Crystals of the new title compound (NH_3_CH_2_CH_2_NH_3_)[CdCl_4_] were obtained as a side product during the preparation of a phosphate in solution. We report here on its crystal structure. Compounds including cadmium chloride and an organic moiety are frequently found in the form CdCl_2_^.^*X*, where *X* is the organic moiety, for example: CdCl_2_^.^CH_5_N_2_S^.^H_2_O (Woode *et al.*, 1987), CdCl_2_^.^CO(NH_2_)_2_ (Furmanova *et al.*, 1996), CdCl_2_^.^NH_2_NHCONH_2_ (Wang *et al.*, 1993), or CdCl_2_^.^2(C_2_H_5_N_3_O_2_) (Cavalca *et al.*, 1960). The title compound, however, contains cadmium in the anionic part of the crystal structure and is isotypic with (NH_3_CH_2_CH_2_NH_3_)[PdCl_4_] (Berg & Sotofte, 1976), (NH_3_CH_2_CH_2_NH_3_)[CuCl_4_] (Birrell & Zaslow, 1972), (NH_3_CH_2_CH_2_NH_3_)[MnCl_4_] (Tichý *et al.*, 1978) and (NH_3_CH_2_CH_2_NH_3_)[NiCl_4_] (Skaarup & Berg, 1978).

Fig. 1 shows [CdCl_6_] octahedra and the 1,2-ethanediammonium cation connected via hydrogen bonds N1-H3···Cl1, N1-H4···Cl2 and N1-H5···Cl2. All chloride ligands of the CdCl_6_ octahedron participate in hydrogen bonding, as well as all hydrogens that are attached to N1.

Packing of (NH_3_CH_2_CH_2_NH_3_)[CdCl_4_] viewed along *a* (Fig. 2) shows a layer of corner sharing [CdCl_6_] octahedra and the neighbouring layer of 1,2-ethanediammonium cations. The minimal Cd—Cd distance within a layer is 5.1747 (8) Å.

The interlayer space is large enough to allow minimal distorsions of the 1,2-ethanediammonium cation molecule, the angles and distances of which have usual values as reported in known compounds containing this cation.

### Experimental

Crystals of the title compound were obtained by mixing solutions of K_4_P_2_O_7_ (10 ml, 0.1*M*), CdCl_2_ (10 ml, 0.1*M*) and three drops of isopropylamine, (CH_3_)_2_(CH)NH_3_. The pH of the resulting solution was controlled with hydrochloric acid (pH = 2.5), stirred for 30 min, and then left to stand at ambient temperatures. After 5 d, colourless crystals appeared that were filtred off and washed with a solution of ethanol-water (80/20). Under the given reaction conditions isopropylamine will not convert into ethylenediamine (en), as evidenced by the structure analysis. Therefore it is most likely that the two supply bottles with isopropylamine and ethylenediamine were confused for synthesis.

### Refinement

All hydrogen atoms were discernible from difference Fourier maps and could be refined to a reasonable geometry. In the last refinement cycles they were nevertheless kept in ideal positions with N—H and C—H distances restrained to 0.87 Å and 0.96 Å, respectively, and with *U*_iso_(H) = 1.2×*U*_eq_ of the respective parent atom.

### Crystal data

**Table:**

(C_2_H_10_N_2_)[CdCl_4_]	*F*(000) = 304
*M**_r_* = 316.3	*D*_x_ = 2.278 (1) Mg m^−^^3^
Monoclinic, *P*2_1_/*c*	Mo *K*α radiation, λ = 0.71073 Å
Hall symbol: -P 2ybc	Cell parameters from 5814 reflections
*a* = 8.6205 (5) Å	θ = 2.8–26.5°
*b* = 7.3425 (8) Å	µ = 3.45 mm^−^^1^
*c* = 7.2937 (7) Å	*T* = 298 K
β = 92.791 (6)°	Irregular shape, colourless
*V* = 461.11 (7) Å^3^	0.27 × 0.13 × 0.08 mm
*Z* = 2	

### Data collection

**Table:**

Oxford Diffraction Gemini diffractometer with Atlas CCD detector	960 independent reflections
Radiation source: X-ray tube	899 reflections with *I* > 3σ(*I*)
graphite	*R*_int_ = 0.023
Detector resolution: 20.7491 pixels mm^-1^	θ_max_ = 26.5°, θ_min_ = 3.6°
Rotation method data acquisition using ω scans	*h* = −10→10
Absorption correction: analytical [implemented in *CrysAlis RED* (Oxford Diffraction, 2008), according to Clark & Reid (1995)]	*k* = −9→9
*T*_min_ = 0.605, *T*_max_ = 0.841	*l* = −9→9
6603 measured reflections	

### Refinement

**Table:**

Refinement on *F*^2^	H-atom parameters constrained
*R*[*F*^2^ > 2σ(*F*^2^)] = 0.010	Weighting scheme based on measured s.u.'s *w* = 1/[σ^2^(*I*) + 0.0004*I*^2^]
*wR*(*F*^2^) = 0.027	(Δ/σ)_max_ = 0.014
*S* = 1.08	Δρ_max_ = 0.13 e Å^−^^3^
960 reflections	Δρ_min_ = −0.13 e Å^−^^3^
44 parameters	Extinction correction: B-C type 1 Lorentzian isotropic (Becker & Coppens, 1974)
0 restraints	Extinction coefficient: 1620 (80)
20 constraints	

### Special details

**Table:**

Refinement. The refinement was carried out against all reflections. The conventional *R*-factor is always based on *F*. The goodness of fit as well as the weighted *R*-factor are based on *F* and *F*^2^ for refinement carried out on *F* and *F*^2^, respectively. The threshold expression is used only for calculating *R*-factors *etc*. and it is not relevant to the choice of reflections for refinement.The program used for refinement, Jana2006, uses the weighting scheme based on the experimental expectations, see _refine_ls_weighting_details, that does not force *S* to be one. Therefore the values of *S* are usually larger than the ones from the *SHELX* program.

### Fractional atomic coordinates and isotropic or equivalent isotropic displacement parameters (Å^2^)

**Table:**

	*x*	*y*	*z*	*U*_iso_*/*U*_eq_	
Cd1	1	0	1	0.01781 (5)	
Cl1	1.03989 (4)	0.20870 (4)	0.71088 (4)	0.02864 (10)	
Cl2	0.70621 (4)	0.05039 (5)	0.96946 (5)	0.02895 (10)	
N1	0.71742 (15)	0.48402 (15)	1.0216 (2)	0.0310 (4)	
C1	0.56378 (15)	0.55139 (19)	0.95400 (19)	0.0286 (4)	
H3	0.789703	0.53995	0.964335	0.0372*	
H4	0.729665	0.504914	1.138852	0.0372*	
H5	0.723446	0.367498	1.001561	0.0372*	
H1	0.55253	0.534368	0.823527	0.0344*	
H2	0.555534	0.678959	0.980677	0.0344*	

### Atomic displacement parameters (Å^2^)

**Table:**

	*U*^11^	*U*^22^	*U*^33^	*U*^12^	*U*^13^	*U*^23^
Cd1	0.01855 (9)	0.01728 (9)	0.01754 (9)	0.00042 (4)	0.00020 (5)	0.00048 (4)
Cl1	0.03678 (18)	0.02466 (16)	0.02474 (16)	0.00197 (13)	0.00416 (13)	0.00987 (12)
Cl2	0.01873 (15)	0.03278 (18)	0.03532 (19)	0.00157 (13)	0.00114 (13)	−0.00058 (15)
N1	0.0225 (7)	0.0334 (7)	0.0369 (7)	−0.0008 (4)	−0.0004 (5)	0.0011 (5)
C1	0.0237 (7)	0.0291 (6)	0.0331 (7)	0.0005 (6)	0.0017 (6)	0.0080 (6)

### Geometric parameters (Å, °)

**Table:**

Cd1—Cl1	2.6427 (5)	N1—H3	0.87
Cd1—Cl1^i^	2.6471 (5)	N1—H4	0.87
Cd1—Cl1^ii^	2.6427 (5)	N1—H5	0.87
Cd1—Cl1^iii^	2.6471 (5)	C1—C1^iv^	1.5169 (19)
Cd1—Cl2	2.5585 (4)	C1—H1	0.96
Cd1—Cl2^ii^	2.5585 (4)	C1—H2	0.96
N1—C1	1.4760 (18)		
			
Cl1—Cd1—Cl1^i^	91.326 (12)	Cl2—Cd1—Cl2^ii^	180
Cl1—Cd1—Cl1^ii^	180	Cd1—Cl1—Cd1^v^	156.050 (14)
Cl1—Cd1—Cl1^iii^	88.674 (12)	C1—N1—H3	109.471
Cl1—Cd1—Cl2	90.766 (12)	C1—N1—H4	109.4712
Cl1—Cd1—Cl2^ii^	89.234 (12)	C1—N1—H5	109.4713
Cl1^i^—Cd1—Cl1^ii^	88.674 (12)	H3—N1—H4	109.4717
Cl1^i^—Cd1—Cl1^iii^	180	H3—N1—H5	109.471
Cl1^i^—Cd1—Cl2	88.066 (11)	H4—N1—H5	109.4711
Cl1^i^—Cd1—Cl2^ii^	91.934 (11)	N1—C1—C1^iv^	110.09 (11)
Cl1^ii^—Cd1—Cl1^iii^	91.326 (12)	N1—C1—H1	109.4717
Cl1^ii^—Cd1—Cl2	89.234 (12)	N1—C1—H2	109.4714
Cl1^ii^—Cd1—Cl2^ii^	90.766 (12)	C1^iv^—C1—H1	109.4709
Cl1^iii^—Cd1—Cl2	91.934 (11)	C1^iv^—C1—H2	109.4709
Cl1^iii^—Cd1—Cl2^ii^	88.066 (11)	H1—C1—H2	108.8487

Symmetry codes: (i) −*x*+2, *y*−1/2, −*z*+3/2; (ii) −*x*+2, −*y*, −*z*+2; (iii) *x*, −*y*+1/2, *z*+1/2; (iv) −*x*+1, −*y*+1, −*z*+2; (v) −*x*+2, *y*+1/2, −*z*+3/2.

### Hydrogen-bond geometry (Å, °)

**Table:**

*D*—H···*A*	*D*—H	H···*A*	*D*···*A*	*D*—H···*A*
N1—H3···Cl1^v^	0.87	2.35	3.2123 (14)	173
N1—H4···Cl2^iii^	0.87	2.46	3.2824 (15)	157
N1—H5···Cl2	0.87	2.34	3.2075 (12)	172

Symmetry codes: (v) −*x*+2, *y*+1/2, −*z*+3/2; (iii) *x*, −*y*+1/2, *z*+1/2.
